# Immature Teratoma in Pregnancy: A Case Report

**DOI:** 10.31729/jnma.8267

**Published:** 2023-09-30

**Authors:** Sapana Amatya Vaidya, Snigdha Rai, Anushree Jha

**Affiliations:** 1 Department of Obstetrics and Gynecology, Paropakar Maternity and Women's Hospital, Thapathali, Kathmandu, Nepal; 2 Kathmandu Cancer Center, Changunarayan, Bhaktapur, Nepal

**Keywords:** *case reports*, *chemotherapy*, *immature teratoma*, *pregnancy*, *surgery*

## Abstract

Immature teratoma is one of the rare malignant germ cell tumours presented in pregnancy. Here, we present 26-year-old pregnant women who had an incidental finding of left adnexal mass in an anomaly scan at 19 weeks of pregnancy. Laparotomy with peritoneal fluid cytology, left salpingo-oophorectomy and omental biopsy at 20 weeks of pregnancy revealed immature teratoma stage 1A, grade 2 in the histopathology report. However, she followed up with the metastatic mass in the pouch of Douglas at 30 weeks of pregnancy in magnetic resonance imaging despite being counselled for possible chemotherapy and surveillance. A baby with a good Apgar score and grade 3 immature teratoma in the metastatic mass was revealed following the exploratory laparotomy and cesarean section at 36 weeks of pregnancy. Fertility-sparing surgery with chemotherapy during pregnancy for high-grade tumours may result in a good prognosis.

## INTRODUCTION

The incidence of malignant ovarian tumours in pregnancy is relatively low with the incidence of 0.2-3.8 cases in every 100,000 pregnancies.^1^ Immature teratoma in pregnancy accounts for less than 1% of all ovarian teratomas.^2^ The five-year survival rate for stage I and II immature teratomas is greater than 90%, 82% for stage III, 72% for stage IV disease and high-grade tumours worsen the prognosis.^3^ Therefore, treatment should be started immediately for the survival benefit even in pregnancy. However, not enough data prevailed on its clinical presentation, diagnosis and management of immature teratoma in pregnancy.

## CASE REPORT

**Figure 1:**
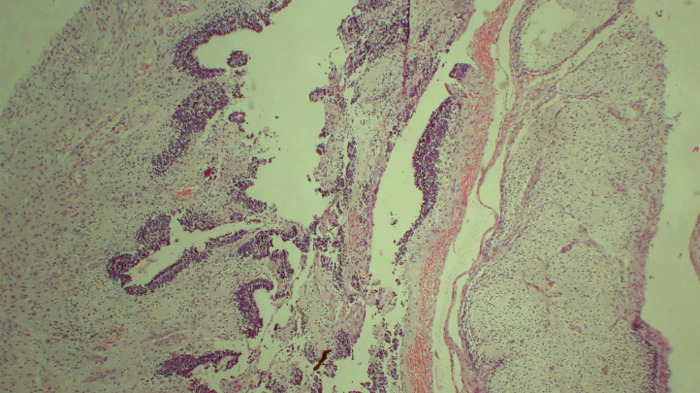
Histopathological photomicrograph showing neuroepithelial component (HE Stain: x100) suggesting immature teratoma of the left ovary.

A 26-year-old pregnant woman with no significant medical history had an incidental finding of a left adnexal mass in an anomaly scan at 19 weeks of pregnancy. The left-sided mass was a large heterogeneous solid cystic mass measuring 12x11x7.5 cm with internal vascularity. She and her family members were counselled about the possible surgery, risk of surgery and outcome based on the ultrasound features, and surgery with possible histopathological diagnosis. As the final diagnosis is always based on the histopathological examination, she underwent laparotomy with peritoneal fluid cytology, left salpingo-oophorectomy, and omental biopsy after a week of diagnosis at Paropakar Maternity and Women's Hospital. Intraoperative findings revealed a left-sided solid cystic mass of about 11x10 cm, the surface was irregular with an intact capsule with a solid cystic area in the cut section. The left tube was stretched over the mass and the right ovary, tube and gravid uterus were normal looking. The histopathology of the specimen was immature teratoma stage 1, grade 2 tumours (Figure 1).

**Figure 2:**
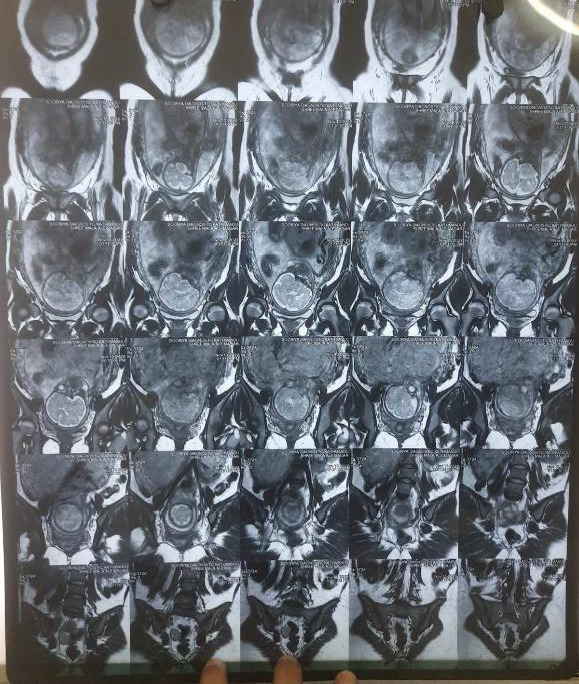
MRI picture of pregnancy with metastatic mass in the pouch of Douglas.

She was referred for medical oncological review following the diagnosis of immature teratoma at Bhaktapur Cancer Hospital. There, she was counselled for magnetic resonance imaging (MRI) and the possible need for chemotherapy. However, only at 30 weeks of pregnancy, she followed up with MRI at Paropakar Maternity and Women's Hospital. MRI revealed a single intrauterine pregnancy with a well-defined altered signal intensity lesion, measuring 5x5 cm with restricted diffusion in the posterior aspect of the right pelvic cavity, presacral region (Figure 2).

Tumour markers, alpha-fetoprotein (AFP) was increased to 945 μg/l (reference range <7.5 μg/l), human chorionic gonadotropin (HCG) was 44,205 mlU/ml, lactate dehydrogenase (LDH) was 220 U/l, carbohydrate antigen-125 (CA-125) was 13.3U/ml and carbohydrate antigen 19-9 (CA 19-9) was <1.4 U/ml.

**Figure 3:**
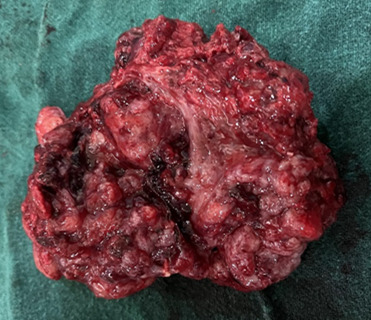
Metastatic mass of pouch of Douglas.

After discussion with the multidisciplinary team (gynecologic oncologist, medical oncologist, pathologist and neonatologist), she was planned for chemotherapy after delivery and excision of possible metastatic lesion at 34 to 36 weeks. The injection of dexamethasone for the lung maturity of the fetus was also planned along with it. After counselling, she underwent exploratory laparotomy with peritoneal fluid cytology, lower segment cesarean section, excision of metastatic deposits along with infracolic omentectomy at 36 weeks of pregnancy at Paropakaar Maternity and Women's Hospital. An alive male of 3 kg with a good Apgar score was delivered. Other intraoperative findings were an encapsulated solid mass of about 8x8 cm with an irregular surface located in the pouch of Douglas (POD) densely adherent to the large bowel posteriorly and posterior surface of the uterus anteriorly (Figure 3).

**Figure 4:**
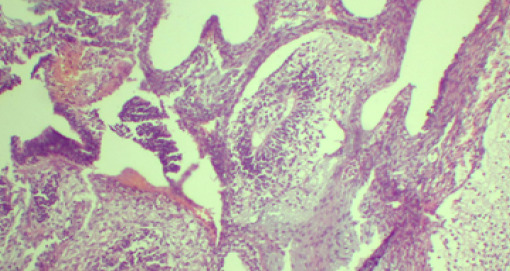
Histopathology of the pouch of Douglas mass: Photomicrograph showing neuroepithelial and rosette component (HE stain: x400) suggesting immature teratoma.

The peritoneal surfaces, undersurface of the diaphragm, liver surface and omentum looked normal. However, the right ovary was not visualized. Histopathology was grade III immature teratoma for the pouch of Douglas mass (Figure 4).

Histopathology of the tissue designated as a separate capsule sent was involved by the tumour but negative for tumour deposits in the omentum. However, the staging was not given due to the limitation of the necessary information.

She was referred back to Bhaktapur Cancer Hospital for chemotherapy. Pre-chemotherapy computed tomography scan of the abdomen and pelvis revealed no abnormality-enhancing lesions in the pelvis and no significant lymph nodes and free fluid in the abdominal and pelvis, no intraabdominal metastasis. She is receiving a BEP (bleomycin, etoposide, cisplatin) regimen of chemotherapy; bleomycin 30 units on days 1, 8, and 15, etoposide 100 mg/m2 for 1-5 days and cisplatin 20 mg/m2 for 1-5 days. After three cycles of chemotherapy of the BEP regimen, a CT scan is planned for further management.

## DISCUSSION

Like other ovarian tumours, immature teratoma in pregnancy is usually asymptomatic, so it is difficult to diagnose based on symptoms, especially during pregnancy. The majority of ovarian tumours in pregnancy are incidental findings during an obstetric ultrasound.^4^ In this case report too, immature teratoma was first diagnosed during a congenital anomaly scan at 19 weeks of pregnancy.

Serum tumor markers in addition to imaging are useful in the diagnosis of immature teratoma in pregnancy. However, pregnancy itself is often associated with elevated serum tumour markers due to the physiological changes it undergoes. Tumour markers like AFP, CEA, LDH, CA-125 and CA19-9 are used for the diagnosis of ovarian tumours. The increasing level of AFP and LDH is often associated with immature teratoma.^5^ In our case, AFP was only raised. Regarding the laterality of the tumour, malignant ovarian germ cell tumours (MOGCT) are generally unilateral except in 10% or the advanced stage with metastasis to the contralateral ovary.^6^ In our case, it was also unilateral which is left-sided.

A multidisciplinary approach should be taken into consideration for the management of ovarian tumours in pregnancy and the therapeutic decision should be based on the histology, grade and stage of the tumour along with the period of gestation. In our case too, a multidisciplinary team approach was done for the management. Immature teratomas with grade 2 or 3 are associated with a greater chance of potentially fatal recurrence predominantly within two years of diagnosis.^7^ Our case with immature teratoma stage I grade 2 with recurrent mass at the pouch of Douglas in MRI at 30 weeks of pregnancy that is within less than six months of diagnosis. Malignant germ cell tumours treated by surgery alone have a poor prognosis that indicates a need for adjuvant chemotherapy.^8^

The most commonly used chemotherapy is BEP (bleomycin, etoposide, cisplatin) used every 3 weeks for 3 or 4 courses and is effective during pregnancy with good neonatal outcomes.^9^ However, our patient did not receive chemotherapy during pregnancy and succumbed to a metastatic mass of grade 3 immature teratoma which could have been prevented by timely instillation of chemotherapy.

Immature teratoma in pregnancy is a rare entity. Fertility-sparing surgery with chemotherapy and surveillance for high-grade immature teratoma (grade 2-3) during pregnancy should be encouraged for a better prognosis. However, treatment during pregnancy for immature teratoma should be based on the gestational age, stage of the disease at diagnosis, fetal risks as a consequence of maternal treatments and the patient's preparedness or desire to continue her pregnancy.
